# Aligning policy objectives and payment design in palliative care

**DOI:** 10.1186/s12904-018-0294-4

**Published:** 2018-03-07

**Authors:** Stephen Duckett

**Affiliations:** Health Program, Grattan Institute, 8 Malvina Place, Carlton, VIC 3053 Australia

**Keywords:** Palliative care, activity-based funding, payment models

## Abstract

**Background:**

Payment models for palliative care vary across nations, with few adopting contemporary payments designs that apply to other parts of the health system.

**Aim:**

To propose optimal payment arrangements for palliative care.

**Approach:**

Review of relevant literature on funding mechanisms in health care generally and palliative care in particular.

**Results:**

Payment models for palliative care should move toward activity-based funding using an agreed classification, be uncapped funding with performance monitoring, and make explicit use of performance metrics and reporting.

**Conclusions:**

If palliative care is to become a universally accessible service, new approaches to funding, based on the experience of funding reforms in other parts of the health system, need to be adopted.

## Background

Palliative care is an increasingly important component of health care provision [1, 2] .Although there are many definitions of palliative care [3, 4], some with competing conceptual bases, common to many is ‘quality of life as a goal of palliative care (and) the inclusion of the family and multidisciplinary practice’ [5]. However, these definitional differences carry across into differences in the definition of ‘palliative care patients’ [6] and information available to patients [7]. More importantly for assessing service planning and access however, is that what ‘good palliative care’ means in practice also still lacks definitional precision [3]. For the purposes of this paper, the policy objective of palliative care is to enhance the quality of life for patients and their carers, and fulfil choices about care style and location, for people who are approaching death.

As people seek more control of their treatment at the end of their lives, consumer expectations of a ‘good death’ , more accurately described as a ‘good dying trajectory’, are challenged in many countries by the poor availability of the necessary palliative care services to facilitate that outcome [8–12], and this is in part driven by funding arrangements which may include co-payments or otherwise limit access to palliative care [13, 14].

Two systematic reviews have concluded that palliative care is cost saving [15, 16] and a further systematic review, updating the literature and broadening the scope of the review, is now underway [17] .Many recent individual studies have also shown palliative care to be cost-effective [18–21], with savings for earlier palliative care greater for patients with mullti-morbidity [19], and different modalities of palliative care provision (consultations vs admission to a designated unit) yielding different incremental cost-effectiveness [22].

Although it is simplistic to view home as always the best place for a person to die [23], the disjunction between preferred and actual location of death [24–26], coupled with the evidence of the cost-effectiveness of palliative care services, the effectiveness of home-based palliative care in facilitating deaths at home [12, 27] and the discrepancy between the number of people receiving palliative care and those that might benefit from it [28], suggests that many countries are underinvesting in these services [14, 29]. Additional investment in palliative care will only be worthwhile if it is appropriately targeted, and if services which are funded are efficient.

It is the purpose of this paper to propose payment arrangements for palliative care which are better aligned to espoused policy objectives for palliative care, subject to the constraint of encouraging efficient care processes. In this paper the term ‘palliative care’ is used to encompass specialist palliative care and hospice services.

### Issues in payment design for palliative care

Countries and jurisdictions differ in the way they pay for palliative care [13, 30, 31], with the inevitable consequence that both providers and consumers face different incentives; and some funding designs may even create perverse incentives, for example by allowing uncapped funding for hospital-based palliative care when home-based care is capped, contrary to the policy direction to encourage home-based care.

Funding design should encourage both allocative (or social) and technical efficiency [32]. In the context of palliative care, allocative efficiency is achieved when there is an appropriate level of investment in palliative care (relative to other investments in alternative areas of healthcare) when there is an appropriate balance between hospital and community or home-based palliative care; and when there is an appropriate level of personal out-of-pocket payments.

A critical aspect of funding system design is therefore ensuring that funding of the different components of the palliative care system is consistent with, and aligned to, the objectives of palliative care policy. Further it should ensure that bottlenecks, distorted incentives and inefficiency through increased provision in high cost vs low cost settings are not created by funding mechanisms [33, 34]. Alignment of policy and funding design will facilitate service delivery implementation by ensuring that internal organisational policies and practices are more likely to be designed consistent with the overall policy goals [35].

#### Appropriate level of investment

The World Health Organisation explicitly includes palliative care in its goal for universal health care, and a recent resolution of the World Health Assembly called on member states to ensure good access to palliative care [36]. Most jurisdictional policies express the same aspiration but, unfortunately, the reality of palliative care provision appears to fall short of that aspiration [37–39], although probably mainly because of the much higher political profile accorded to acute care, it may also be due to ‘conventional health economic analyses … undervaluing the benefits derived by society from the provision of palliative care’ [40]. These aspirational goals need to be achieved within the context of both constrained public funding and ensuring value for money.

Funding policy design needs to be informed by two critical considerations to ensure value for money: the risk of supply- and demand-side moral hazard and the risk of provider adverse selection [41].

Supply-side moral hazard involves providers delivering more services than consumers require. It is particularly relevant in fee-for-service funding environments, where providers are reimbursed for each additional service [42, 43], and is especially a risk in environments, such as the United States, with a large corporate for-profit sector [44]. Contemporary payment system design in health care balances payer and provider risk: fee-for-service payment involves the greatest payer risk and the least provider risk and, for this reason, payers are replacing fee-for-service reimbursement by funding strategies which ‘bundle’ payments either over time – known as ‘capitation’ approaches – or across an episode of care using some form of classification of the episode [45].

Adverse selection occurs when a payer (such as an insurance company) or a provider has a disproportionate share of ‘bad risks’ –patients who have greater than expected health care costs. Adverse selection is mitigated by use of risk adjustment in either episode payment or capitation formulae [46]. The corollary of adverse selection is ‘cream skimming’ which involves providers’ selecting patients who are expected to have lower needs than the reimbursement formula predicts [47]. This is a risk either because the provider has access to additional information about the patient not captured in the risk-adjustment formula [48] and/or because of behavioural responses by consumers [49]. Again, the risk can be mitigated, but not eliminated, by good risk adjustment and limiting the ability of palliative care providers to select patients, for example, by implementing geographic assignment of patients.

In budget-constrained systems, administrative rules might be used to limit access to palliative care, such as limiting access to those with an anticipated life expectancy of six months or less [50]. Crude rules such as this take no account of different illness trajectories and so may discriminate against those with severe, on-going illness [51], create ethical issues for the clinicians involved as gatekeepers [52], rely on potentially inaccurate estimates of life expectancy [53, 54] and undermine a more holistic vision for the role of palliative care [55]. They are especially cruel if accompanied by limitations on the extent to which patients receiving palliative care can also receive intensive disease–directed treatments such as blood transfusions, which may be aimed at improving quality of life [56]. Rules put in place in the United States may have been developed because of the absence of universal coverage for substitute services such as residential (nursing home) care. However, the significant increase in spending on hospice care in the United States has been driven by an increase in the proportion of decedents who have received hospice services rather than by spending per patient which has been constant [57], and so can be interpreted as an objective of policy not as a cost ‘blow out’.

In countries with universal health care, there seems to be no justification to limit access to palliative care services to people who expect to die within a defined period and so the risk of demand-side moral hazard (e.g. substitution from other forms of care) is lower. It is unlikely that people would seek palliative care unless the prospect of death were high and so demand for these services is naturally capped to those who expect to die in the near future. If funding for palliative care services were uncapped, supply-side moral hazard could be mitigated using performance metrics e.g. that 90 per cent of clients managed by a palliative care service died within x months of admission to care. Components of funding could still be capped to encourage desirable policy directions e.g. hospital funding could have tighter caps than out-of-hospital funding if the policy directions were to encourage dying outside hospital.

Palliative care as a distinct and recognised service is a relatively recent newcomer to health care – in the United Kingdom it is often dated to the pioneering work of Dame Cicely Saunders in the 1960s and 1970s, with its emergence in the United States dating from the same period [58–60]. With advances in medical technologies, and ever increasing opportunities for intervention, the challenge of developing and meeting the appropriate goals of care [61] becomes greater and the place of care becomes even more important. The result appears to be that the supply of palliative care lags behind need and demand [37–39].

The importance of ensuring appropriate funding design is increased as countries seek to expand palliative care, to meet the universality aspiration. Inappropriate funding design may mean that additional palliative care investment is not used as efficiently as possible that is, funding increases do not generate a commensurate increase in the extent to which palliative care needs are met.

#### Balancing hospital and home care

A second aspect of allocative efficiency in palliative care is ensuring the appropriate balance of investments in supporting home vs hospital-based palliative care programs. There is an imbalance in actual compared to preferred location of death in most countries, with most people preferring a death at home [62, 63] but with most deaths occurring in institutional settings [64].

Importantly, better palliative care or other supports can influence the likelihood of death in the preferred setting [26, 27, 65], and the length of time at home even if death occurs in hospital [66]. As indicated above, in addition to reflecting people’s priorities better, increasing the proportion of deaths occurring at home could also improve technical efficiency [15, 16].

Funding policies can inadvertently preference one type of care over another. In Belgium, for example, basic palliative care is funded through uncapped social insurance funding but specialist, hospital-based palliative care is block funded leading to overwork issues [34]. This could potentially lead to problems of gaining access to palliative care.

In Victoria, Australia, hospital-based palliative care is funded under that state’s activity based funding arrangements, so each additional hospital admission attracts additional funding (although overall hospital funding, across all types of patients, is capped). In contrast, community or home based palliative care is funded on a population basis, so additional patients do not attract additional funding. The different funding mechanisms thus create a greater incentive for admissions to hospitals than for community-based treatment.

#### Personal out-of-pocket costs

There are two main theoretical justifications for personal out-of-pocket costs in health care. The first is demand-side moral hazard: that unless there is a personal contribution, patients will consume too much of the ‘free’ service. Co-payments reduce the demand for non-institutional services (such as general practitioners and pharmaceuticals) but not for hospitals, and have a higher impact on low income people [67]. Aside from the problematic equity issues, such co-payments may thus perversely encourage hospital, rather than community-based palliative care options [14], even more so if co-payment policies lead to higher out-of-pockets for home-based care. In the context of freely available substitutes, as is the case in most countries with universal health care, the risk of demand-side moral hazard is low. Together these factors would argue against significant use of patient co-payments.

The second justification for co-payments is normative: that the alternative to co-payments is additional collective funding - either through taxation or social insurance – and that taxes and social insurance contributions need to be minimised because of the ‘dead weight’ burden of taxation [68]. The level of taxation is a social choice and perceptions of the appropriate level of taxation will differ across countries and the perceived purposes of taxation. There is a strong ethical argument for public financing of health care [69] ,with end-of-life care potentially having an even stronger ethical justification for public funding.

### Contemporary options for payment design for palliative care

Historically health services were funded on the basis of negotiated (or ‘block’) payments, standard payments for each day of stay (‘per diems’), or fee-for-service. These types of payments have intrinsic weaknesses – the first incorporating no incentives for efficiency, the second not accounting for variation in patient need, and the third involving a high risk of supply-side moral hazard.

There are two main contemporary options for palliative care payments: activity-based payments and population (or capitation) based payments [45]. Both involve some level of bundling of services into a single payment. Increasingly, both these options are often supplemented by performance-related payments, despite the weak evidence basis for this policy direction [70]. Activity-based payments promote equity in that they ensure that like patients are funded alike [71].

#### Activity-based payments

Activity-based funding involves paying for health services according to a patient classification system which groups patients into clusters of patients expected to have a similar resource profile and which themselves are clinically meaningful [72] .Although initially developed to describe acute inpatient care, classification systems have now been developed for a range of types of health care [73].

A number of classification systems to describe palliative care patients either have been or are being developed, the most comprehensive being the Australian National Sub-acute and Non-acute Patient classification which has a palliative care sub-classification [74]. NHS England has proposed using a classification analogous to Australian National Sub-acute and Non-acute Patient classification [75].

The current version of the Australian classification (version 4, available at https://www.ihpa.gov.au/what-we-do/subacute-and-non-acute-care accessed 18 February 2018) divides palliative care patients into admitted and non-admitted branches, then into adult and paediatric branches. Patients are then split by ‘phase of care’: stable, unstable, deteriorating and terminal [76]. Further splits are made according to other measures such as ability to perform activities of daily living.

The classification has 20 admitted patient groups (14 adult, 6 paediatric) and 14 non-admitted groups (9 adult, 5 paediatric; with the unit of grouping being the phase of care [77]), although prices have only been assigned to some of the groups of the classification. The current relative prices (in A$) for each of the adult groups which have been assigned prices are shown in Table 1.

**Table 1:** Prices established for palliative care for normal (‘inlier’) palliative care inpatients, Australia, 2017/18

Australian National Sub-acute and Non-acute Patient classification class	Price (A$)
Stable phase, RUG-ADL 4-5	$ 8,354.86
Stable phase, RUG-ADL 6-16	$ 8,948.48
Stable phase, RUG-ADL 17-18	$ 8,385.30
Unstable phase, First Phase in Episode, RUG-ADL 4-13	$ 2,148.13
Unstable phase, First Phase in Episode, RUG-ADL 14-18	$ 1,903.61
Unstable phase, Not first Phase in Episode, RUG-ADL 4-5	$ 2,955.82
Unstable phase, Not first Phase in Episode, RUG-ADL 6-18	$ 2,184.46
Deteriorating phase, RUG-ADL 4-14	$ 5,980.87
Deteriorating phase, RUG-ADL 15-18, Age >= 75	$ 4,261.39
Deteriorating phase, RUG-ADL 15-18, Age 55-74	$ 4,458.77
Deteriorating phase, RUG-ADL 15-18, Age <= 54	$ 6,225.88
Terminal phase	$ 3,040.27
Adult Same-Day Palliative Care	$ 977.58

1. RUG-ADL is Resource Utilisation Groups-Activities of Daily Living score

2. The prices shown are those that apply for Commonwealth Government payments under Australia’s federalism arrangements, payments are supplemented for patients who are indigenous or live in remote Australia, or both

Source: Derived from https://www.ihpa.gov.au/publications/national-efficient-price-determination-2017-18 accessed 18 February 2018

All activity classifications – and the Australian palliative care classification is no exception - assume homogeneous quality, an assumption belied in practice. Although it has been shown that it is possible to incorporate quality of care measures into an efficiency measure [78, 79], generally quality variation in practice has been addressed in payment policies through supplements or ‘pay for performance’ incentives, as discussed below.

An alternative activity-based funding approach is to pay for each death managed. This has the advantage of simplicity and would create a strong incentive on the palliative care organisation to manage care in the least cost setting. Such a model may enhance continuity of care and recognise that patients’ needs evolve over their end-of-life path.

Unfortunately, there is no published evidence of such a system in practice, nor has a risk-adjustment model been developed to recognise the different costs involved in managing different types of patients (e.g. paediatric patients compared to adult, patients with different levels of carer support, and patients with different family circumstances such as experiencing domestic violence or family estrangement, or with different levels of acceptance of the prognosis). However, it is possible that such a model could be developed for larger organisations that could manage the risk of patient variation. Performance benchmarks would also need to be developed to mitigate the risk of under-servicing by providers.

#### Capitation payments

Payments based on the population covered provide incentives to keep populations healthy and manage care in the most appropriate setting [45]. Well-designed capitation payments could be used to address issues of geographic equity [48, 80]. Capitation (or per member per month) payments are common in a number of countries and generally cover all health care needs. However, in the United States, where capitation payments are a common payment approach, Medicare population-based funding arrangements ‘carve out’ hospice programs which are reimbursed on the typical mix of fee-for-service and per diem payments [81].

Specialist palliative care organisations could be paid on the basis of the population served and the expected number of deaths in that population. However, again there is no published risk-adjustment formula for such a payment model. As with a ‘per death’ activity-based model, a capitation based model involves a risk of underservicing, as there is no incentive on the organisation to maximise referrals or reach out to populations known to have a low take-up of palliative care [82]. Depending on how capitation rates are determined, it may enshrine historic inequitable service access.

Further, earlier involvement of palliative care teams leads to better outcomes [83–88], leading to heterogeneity in palliative care activity as a result of the variability in length of life after initial involvement. In turn this creates heterogeneity in costs for capitated organisations and, because risk adjustment requires developing homogeneous categories, makes it harder to develop an adequate formula to set a fair capitation payment for providers.

Finally, in the absence of good measurement of the extent to which a population’s palliative care needs are met, if the palliative care experience is similar to primary care, capitation funding is likely to lead to lower levels of productivity and efficiency than activity-based funding [43, 89–91].

#### Performance-based supplements

Many countries and health systems are adopting add-on or bonus payments to reward better performance on policy-relevant metrics [45, 92]. Often referred to as ‘pay-for-performance’, some are more accurately described as paying providers for following preferred processes (such as referrals to specialists) or paying for additional data collection. The evidence about whether pay-for-performance works in practice is quite mixed [93–97], although generally out-of-hospital programs which involve larger rewards or focused on process measures seem to be more likely to have positive impacts [98].

Poorly designed and conceptualised pay-for-performance can have quite negative impacts on care. The English experience with the Liverpool Care Path is an unfortunate one: what was proposed initially as a way of improving end of life care, became an impersonal, politicised and discredited approach which was abandoned [99–104]. If pay-for-performance were to be incorporated into palliative care funding arrangements, it should adopt ethical design principles [105] and be rigorously evaluated [106].

Regardless of whether they are used in pay-for-performance arrangements, there are a number of palliative care measures which should be used in benchmarking or as contract performance criteria, including the Palliative Care Outcome Scale [107], and other proposed measures of palliative care impact which better account for the value of palliative care to families and carers [108, 109]. National benchmarking organisations such as the United States National Palliative Care Registry (see https://registry.capc.org 18 February 2018) or the Australian Palliative Care Outcomes Collaboration (see http://www.pcoc.org.au/ accessed 18 February 2018) already collect and report on many such indicators. Appropriate measures might include measures of timeliness of responding to initial referrals and to requests for assistance (both in normal business hours and out-of-hours), emergency department visits in the last six months of life, whether expectations of place of care and place of death have been realised; and patient reported outcome measures [108, 110–115].

Performance supplements should not only provide incentives for appropriate use of services, or meeting expectations of place of death, but should also address the all-important question of style of care provision. Patients consistently report that *how* care is provided to them is critical [116], and integrated care - care which ensures continuity - should be measured and valued in performance metrics [117–120].

Over time, these measures could be used in the design of pay-for-performance supplements.

#### Interaction of palliative care and other treatments

Early referral to palliative care is beneficial [83], in these circumstances palliative care should not be an alternative to curative treatment, but at least in the early stages, should be a complement. This will change over the course of the illness trajectory as the goals of treatment and care change [61].

Integration of services with a curative intent and provision of palliative care services involves change to contemporary service provider structures and processes [39, 121], made more difficult because of the multiple care settings involved in palliative care [66] cultural differences between different types of care and health system fragmentation [38]. Payment for palliative care should recognise the complementary nature of palliative care services and services with a curative intent, especially at the early stages of the disease trajectory.

Early involvement of palliative care in the disease trajectory will often mean that discussions about treatment choices and planning about palliative care will take place simultaneously with treatments with a curative intent. If there are separate funding streams for curatively-oriented and palliative care services, consideration will need to be given to how they mesh together.

There are two basic options. First, the two different funding systems could be designed and implemented in parallel, complementary funding existing side-by-side. In community or hospital-based ambulatory care, separate activity-based payments could be made for curative intent and palliative care services, even for overlapping payment periods (e.g. if a 30-day window is used as the payment episode. This approach has the advantage of simplicity.

The second, alternative approach, is for integrated funding -with some form of bundling of the palliative and curatively-oriented components of care. This would provide stronger incentives for care continuity between palliative and curative providers, albeit at the possible expense of administrative complexity. Integrated funding would probably stimulate discussions and negotiations about how curative-intent and palliative care teams will work together and how leadership will shift as the goals of care evolve [61]. In addition to the benefit that improved clarity of roles will bring to patient care, such discussion and negotiation will serve the pragmatic purpose of resolving how funding will be divided and also who will be accountable for which performance metrics (and how joint accountability will be handled). Bundled payment of this kind is a desirable direction for funding design in the medium to longer term.

An important interaction is between palliative care payment policy and payment arrangements for nursing homes and other residential aged care facilities [14]. A high proportion of older people die in aged care facilities [25, 64, 122], and the converse is also true, that the mortality rate in nursing homes is high, a natural consequence of the age and frailty of nursing home residents. Importantly, the risk of death in a nursing home can be predicted reasonably well [123]. Death in a nursing home is ‘normal’, and addressing the palliative care needs of residents, such as improved emotional and spiritual care, help with personal cleanliness, and treatment for pain [124], should be core business of the nursing home.

Depending on overall service system design, the additional services for dying patients might be incorporated in routine nursing home funding formulae, either as part of the needs-based funding or as a ‘death supplement’; provided by additional specialist palliative care services by community palliative care agencies and funded through that mechanism; or a combination of both approaches.

For inpatient episodes, standard care-bundling rules that are already used for activity-based funding would apply.

An important contemporary care issue is the provision of clinically futile care, or more accurately, non-beneficial care: care which has no reasonable expectation of leading to survival outside an acute setting [125–127]. Greater clarity about the goals of care [61], involvement of palliative care services in treatment decisions at an early stage, and inclusion of measures of inappropriate care at the end-of life [114, 128], may all help to reduce the incidence of futile care. Although current economic incentives may play a role in perversely encouraging futile care [129], a host of other factors are also involved [130, 131]. In terms of palliative care payment design, the objective should be to encourage a ‘goals of care’ approach, although this may be better facilitated by organisational processes rather than financial incentives.

## Discussion

Groeneveld et al. have identified six ‘desirable features’ funding models for palliative care should incorporate [13]. These comprise supporting early access to palliative care (not just at the end of life); an appropriate mix of services with palliative and curative intent; services in the most appropriate location; avoidance of financial hardship to service users and families; stable and predictable funding that allows services to be planned and developed in a coherent way; and service arrangements with clear entitlements, and that are easy to understand and navigate, and avoid unnecessary administration and transaction costs.

As discussed in this paper, in order to achieve those desirable features, funding models will need to evolve along the lines shown in Table 2.

**Table 2:** Desirable funding model direction for palliative care

Current models	More desirable models
Per diem or fee-for-service payment	Activity-based funding using an agreed classification such as AN-SNAP
Capped funding	Uncapped funding with performance monitoring
Separate funding mechanisms for different sites of care or death	Funding arrangements designed in light of policy objectives to encourage death in preferred location. Funding could be separate for different sites but must be aligned to reduce incentives on providers to choose site of care inappropriately and to ensure policy objectives are achieved
Regular use of out-of-pocket payments	Reduced use of out-of-pocket payments
No, or implicit, performance metrics	Explicit use of performance metrics and reporting, potentially including use as part of a pay for performance program

Prospective payment for acute inpatient care using activity-based funding was introduced in the United States for Medicare patients more than 40 years ago and has since been adopted in most publicly funded health systems, especially in high-income countries [132–134]. In contrast, there is no dominant funding model for palliative care [13], with many jurisdictions following idiosyncratic approaches with little policy learning across national boundaries.

There is a gap between needs for and supply of palliative care in many countries. Palliative care services are beginning to move beyond their cancer care origins to address needs more broadly defined [28], which increases the gap between this now more broadly defined need and supply. Although palliative care is still a small proportion of health budgets, growing provision will place a challenge on funders. Some resources may be released through improved efficiency in cost per visit, or in terms of a more efficient care path to death – fewer emergency department visits or reduced use of intensive care unit beds as examples – but additional resources may still be required.

If palliative care is to become a universally accessible service, new approaches to funding, based on the experience of funding reforms in other parts of the health system, need to be adopted. However, the funding models used in acute in-patient care need to be adapted to take account of the unique nature of policy objectives for palliative care. As palliative care policies are redeveloped, funders should give explicit consideration to adopting new funding approaches as canvassed here to ensure funding policy and aspirational service objectives are aligned. Because policy objectives and contexts are country dependent, palliative care funding models will also differ across nations but hopefully will still allow for cross-national learning.

## Conclusion

In conclusion, what has been argued in this paper is that palliative care decision-makers need to ensure that they assess the full range of potential palliative care funding designs, including assessing potential perverse incentives caused by their choices. The literature on palliative care funding design is still sparse. This provides an additional reason for policy makers to encourage reporting into the public domain of the design adopted and evaluations of the impact of new palliative care funding models.

## Abbreviation

RUG-ADL: Resource Utilisation Groups-Activities of Daily Living score
